# Economic Factors Associated with AI Adoption in Oncology: Cost-Effectiveness Perceptions, Reimbursement Readiness, and Return-on-Investment Confidence Among Romanian Healthcare Professionals

**DOI:** 10.3390/healthcare14172820

**Published:** 2026-09-02

**Authors:** Dragoș-Ciprian Negoiță, Livia Stanga, Horia Silviu Branea, Ciprian Ilie Roșca, Adrian Cosmin Ilie, Ovidiu Rosca

**Affiliations:** 1 Doctoral School, Faculty of Medicine, “Victor Babes” University of Medicine and Pharmacy Timisoara, Eftimie Murgu Square 2, 300041 Timisoara, Romania; dragos.negoita@umft.ro; 2 Discipline of Microbiology, Faculty of Medicine, “Victor Babes” University of Medicine and Pharmacy Timisoara, 300041 Timisoara, Romania; stanga.livia@umft.ro; 3 Department of Internal Medicine I—Medical Semiotics II, Faculty of Medicine, “Victor Babes” University of Medicine and Pharmacy Timisoara, 300041 Timisoara, Romania; 4 Department V, Internal Medicine I—Discipline of Medical Semiology I, Center of Advanced Research in Cardiology and Hemostaseology, Faculty of Medicine, “Victor Babes” University of Medicine and Pharmacy Timisoara, 300041 Timisoara, Romania; 5 Department of Functional Sciences, Discipline of Public Health, Center for Translational Research and Systems Medicine, “Victor Babes” University of Medicine and Pharmacy Timisoara, 300041 Timisoara, Romania; ilie.adrian@umft.ro; 6 Department of Infectious Diseases, “Victor Babes” University of Medicine and Pharmacy Timisoara, 300041 Timisoara, Romania; ovidiu.rosca@umft.ro

**Keywords:** artificial intelligence, medical oncology, cost–benefit analysis, healthcare economics and organizations, reimbursement mechanisms

## Abstract

Background and Objectives: Artificial intelligence (AI) tools promise efficiency gains in oncology, yet adoption depends on economic factors that remain under-characterized in Eastern European health systems. We quantified AI economic literacy, cost-effectiveness perceptions, reimbursement readiness, and return-on-investment (ROI) confidence among Romanian oncology professionals; we described candidate economic adoption profiles and examined whether sector was associated with the strength of the indirect association between literacy and willingness to invest via ROI confidence. Materials and Methods: A multicenter cross-sectional survey (*N* = 108) was conducted between September 2025 and April 2026 at “Victor Babes” University of Medicine and Pharmacy Timisoara and affiliated oncology services. Participants completed a 25-item AI Economic Literacy Index (AIELI; 0–25) plus 1–5 scales for ROI confidence, willingness to invest, perceived financial barriers, cost-effectiveness perception, and adoption intention. Analyses used Spearman correlations, multivariable logistic regression, k-means clustering, and covariate-adjusted moderated mediation with 5000 bootstrap resamples. Results: Mean age was 41.3 ± 10.7 years; 58.3% were female. AIELI was moderate (13.7 ± 4.6/25). Familiarity favored cost-effectiveness analysis (59.3%) over AI-specific reimbursement codes (16.7%). High willingness to invest occurred in 48.1% and was independently associated with higher AIELI (aOR 1.78 per +1 SD; 95% CI 1.17–2.71), higher ROI confidence (aOR 1.93; 1.24–3.02), lower perceived financial barriers (aOR 0.58; 0.37–0.91), and prior AI training (aOR 2.34; 1.13–4.86). Three exploratory profiles were identified: Cost-Conscious Adopters (*n* = 43), Reimbursement-Cautious (*n* = 37), and Budget-Constrained Skeptics (*n* = 28). Moderated-mediation models were consistent with a sector-conditional indirect association, largest in private clinics (β = 0.193; 95% CI 0.087–0.318) and weakest in public hospitals (β = 0.072; 0.014–0.158). Conclusions: In this exploratory cross-sectional sample, AI economic literacy and ROI confidence were associated with willingness to invest in oncology AI, interpreted as stated support for investment rather than an enacted procurement decision, since many respondents lacked formal budgetary authority. Because the design cannot establish temporal ordering, whether sector-tailored capability-building and reimbursement clarity would increase adoption remains a hypothesis for prospective evaluation.

## 1. Introduction

Cancer care has become one of the most economically intensive domains in modern medicine, driven by the rising incidence of malignant disease, the rapid translation of high-cost targeted therapies and immunotherapies into routine practice, and the growing complexity of multimodal treatment pathways. Health systems across Europe report sustained year-on-year increases in oncology expenditure, with drug acquisition, imaging, and inpatient stays accounting for the largest fractions of total spending [1]. Within this environment, artificial intelligence (AI) has emerged as a candidate technology for cost containment and value generation: applications include AI-assisted radiologic interpretation, treatment-response prediction, dose optimization in radiation oncology, drug-dosing personalization in chemotherapy, and administrative automation. Each of these touches a distinct cost center, and each carries different evidence requirements, procurement processes, and reimbursement implications [2,3]. The economic case for clinical AI is therefore not monolithic; it depends on application domain, organizational context, and the willingness of payers and providers to internalize the up-front and recurring costs of these technologies.

Despite a growing literature on the clinical performance of oncology AI, evidence on its economic determinants of adoption remains comparatively sparse and methodologically heterogeneous. Cost-effectiveness analyses (CEA) of AI in cancer screening, pathology, and decision support have yielded mixed conclusions, often constrained by short time horizons, uncertain model durability, and limited real-world data on implementation costs [4,5]. Budget impact analyses (BIAs), which are arguably more relevant to hospital decision-makers because they capture short-term affordability rather than long-term efficiency, are similarly underdeveloped for AI products [6]. Health technology assessment (HTA) agencies have only recently begun issuing AI-specific methodological guidance, and few jurisdictions have established dedicated reimbursement codes for AI-enabled clinical services [7]. As a result, clinicians, administrators, and pharmacists often confront procurement and deployment decisions without standardized economic templates, increasing reliance on subjective ROI estimates and intra-institutional negotiations.

A growing body of implementation science suggests that the gap between economic potential and real-world adoption is shaped by both organizational readiness and individual-level factors, including knowledge, confidence, and perceived burden [8,9]. In oncology, where cost decisions intersect with high-stakes clinical responsibility and constrained reimbursement, professionals’ economic literacy—that is, their understanding of cost-effectiveness, budget impact, and reimbursement mechanisms—may directly influence whether AI tools are recommended, requested, or resisted [10]. Yet, most existing AI-acceptance surveys focus on perceived usefulness, ease of use, and trust, with limited attention to economic competence. This omission is consequential because economic reasoning underpins many tangible adoption decisions: whether to allocate capital budget to an AI-imaging platform, whether to negotiate a value-based contract, or whether to advocate for a new reimbursement code at the national level [11].

Reimbursement readiness merits particular scrutiny in oncology AI. Unlike traditional medical devices, many AI tools function as continuously updated software services, raising novel questions about how to price recurring fees, who bears the cost of model maintenance and re-validation, and whether existing diagnosis-related group (DRG) payments adequately cover AI-enabled care [12]. In several European systems, the absence of AI-specific tariffs has led to ad hoc cost-bundling within existing oncology procedure codes, a practice that can disincentivize investment by obscuring true economic value [13]. Furthermore, the recently operationalized EU Artificial Intelligence Act adds compliance costs—documentation, monitoring, conformity assessment—that further complicate ROI calculations [14]. Understanding how clinicians and administrators perceive these reimbursement realities is essential to designing interventions that align professional incentives with sustainable AI implementation.

Romania provides a particularly informative setting to examine these dynamics. The Romanian oncology system is structured around regional public hospitals operating under fixed budgets negotiated with the National Health Insurance House (CNAS), supplemented by a growing private oncology sector and academic centers engaged in research and innovation. Reimbursement is governed by the Romanian Agency for Medicines and Medical Devices (ANMDMR), with HTA processes that have historically focused on pharmaceutical products rather than digital health technologies [15]. Capital expenditure on imaging and informatics is concentrated in tertiary public hospitals and private chains, while smaller institutions face acute affordability constraints. These heterogeneities create marked variation in how AI economic reasoning is operationalized day-to-day, making Romania a useful case study for the broader Eastern European context where similar structural conditions prevail [16].

Three strands of the recent literature frame the present analysis and clarify what is, and is not, already known. First, health economic evaluations of clinical AI have expanded rapidly but remain methodologically uneven: recent syntheses report incomplete costing of implementation, integration and model-maintenance resources, short analytic horizons, and a predominance of low-maturity technologies, which together limit the usefulness of the existing evidence base for reimbursement decisions [17,18]. Second, work on payment models for digital health has begun to move away from device-style one-off reimbursement towards subscription, per-use, and outcome-linked arrangements while documenting the scarcity of dedicated AI tariffs outside a small number of jurisdictions [12,13,19]. Third, a family of survey instruments now measures digital readiness and eHealth literacy among clinicians and citizens, including the eHealth Literacy Scale and the Digital Health Literacy Instrument [20,21]; these instruments capture the capacity to find, appraise, and apply digital health information, but none assesses knowledge of the economic and reimbursement architecture that determines whether an AI tool can be purchased and sustained. Studies of eHealth and AI adoption in Central and Eastern Europe, including Romanian analyses grounded in the Technology Acceptance Model and surveys of physicians’ perceptions of AI, have concentrated on perceived usefulness, ease of use, trust, and general knowledge deficits rather than on cost-effectiveness reasoning, budget impact, or payment infrastructure [22,23]. The present study is positioned in that gap; it is not a further examination of generic acceptance constructs.

In this cross-sectional study, we measured AI economic literacy, cost-effectiveness perceptions, reimbursement readiness, ROI confidence, perceived financial barriers, and willingness to invest among Romanian oncology professionals. Our objectives were threefold: (i) to quantify the level of and variation in these constructs across roles, sectors, and cancer-care contexts; (ii) to describe data-driven economic adoption profiles using unsupervised clustering and to characterize their structural and behavioral correlates; and (iii) to estimate a moderated-mediation model in which AI economic literacy is indirectly associated with willingness to invest through ROI confidence, with sector examined as a first-stage moderator of that indirect association. We hypothesized that (a) economic literacy and ROI confidence would be the strongest correlates of investment willingness, (b) financial barriers would inversely relate to adoption intention, and (c) the indirect association of literacy through confidence would be largest in private clinics, where capital decisions are more directly linked to professional behavior, and smallest in budget-fixed public hospitals.

The intended contribution of this work is therefore fourfold. (i) Setting: to our knowledge, this is the first survey to characterize the economic—rather than attitudinal—determinants of AI adoption specifically within oncology, a discipline in which capital-intensive imaging, high-cost systemic therapy, and DRG-coded activity intersect within a single cost center. (ii) Context: it is situated in an Eastern European, CNAS-financed system in which HTA has historically been oriented towards pharmaceuticals, a structural configuration that differs materially from the Western European and North American settings in which most AI-acceptance surveys have been conducted. (iii) Construct: it introduces, and documents the preliminary development of, an objective knowledge measure of AI-specific health-economic literacy, complementing existing self-report digital-readiness instruments that contain no economic content. (iv) Model: it specifies a covariate-adjusted moderated-mediation model in which sector is treated as a first-stage moderator, allowing the strength of the literacy–confidence–investment association to be compared across financing environments. Consistent with the cross-sectional design, all analyses are exploratory and hypothesis-generating: this study characterizes associations and is intended to inform the design of future longitudinal and interventional work, not to establish causal or temporal ordering.

## 2. Materials and Methods

### 2.1. Study Design and Setting

We conducted a multicenter cross-sectional survey to examine the economic determinants of AI adoption among healthcare professionals involved in oncology care in western Romania. This study was coordinated by the Department of Health Economics and Pharmacoeconomics and the Department of Medical Oncology, Faculty of Medicine, “Victor Babes” University of Medicine and Pharmacy Timisoara, in collaboration with the OncoGen Center for Translational Oncology Research at the “Pius Brinzeu” Clinical Emergency County Hospital, Timisoara. Recruitment took place over an eight-month period between September 2025 and April 2026, encompassing one full budgetary cycle so that respondents were exposed to both procurement-planning and end-of-year reconciliation phases. This study targeted three structurally distinct sectors—public hospitals operating under CNAS-financed fixed budgets, private oncology clinics with mixed insurance and out-of-pocket revenue streams, and academic centers with research-derived income—to capture heterogeneity in economic decision-making contexts. Six institutions participated: two public hospitals providing oncology services, three private oncology clinics, and one academic center (“Victor Babes” University of Medicine and Pharmacy Timisoara, together with the affiliated OncoGen Center at the “Pius Brinzeu” Clinical Emergency County Hospital). All participating sites are located in western Romania. Respondents were therefore clustered within a small number of institutions; because six clusters are too few to support stable random-effects estimation, no multilevel adjustment was applied, and the resulting possible understatement of standard errors is acknowledged in Section 4.2. Recruitment was confined to one region of the country, and the consequent potential for regional selection bias is treated as a limitation rather than as a feature of the design.

The survey was designed to reflect end-user, governance-facing, and pharmacy/finance perspectives because oncology AI procurement and use require coordinated input across clinical, pharmaceutical, administrative, and health-economic functions. Reporting follows STROBE-aligned conventions for cross-sectional observational studies. Participation was voluntary, anonymous, and uncompensated. This study was conducted in accordance with the Declaration of Helsinki and approved by the institutional review board of “Victor Babes” University of Medicine and Pharmacy Timisoara prior to data collection. No identifying information was retained in the analytic dataset. A pilot phase involving 14 oncology professionals (not included in the final analysis) was used to refine item wording, response burden, and survey flow; pilot data informed item-level revisions to, and the removal of two items from, the AIELI knowledge test; the development, expert review, cognitive pretesting, and preliminary psychometric evaluation of the instrument are reported in Section 2.3.1.

### 2.2. Participants and Sampling

Eligible participants were adults (≥18 years) currently employed in a role involving direct or indirect oncology care delivery, oncology-related health economics, or oncology pharmacy at one of the participating institutions. We targeted six professional groups in approximate proportion to their representation in regional oncology services: medical oncologists, radiation oncologists, oncology nurses, hospital pharmacists, administrators/managers responsible for oncology cost centers, and health economists with experience in HTA or pharmacoeconomic evaluation. Exclusion criteria were as follows: (i) incomplete electronic informed consent; (ii) missing responses preventing computation of the primary outcome (willingness to invest); and (iii) duplicate submissions identified via timestamp and response-pattern analysis.

Recruitment used a hybrid strategy combining department-level invitations distributed through institutional email channels, in-person announcements at oncology multidisciplinary tumor board meetings, and snowball sampling within the regional oncology professional network. A total of 124 individuals opened the survey link; 116 provided initial consent; 108 produced analyzable responses (8 exclusions: 4 incomplete consent or submission, 3 with >25% missing items on key economic constructs, and 1 duplicate). Departmental invitations were distributed to an estimated 240 eligible professionals across the six participating sites, giving an approximate link-opening rate of 51.7% (124/240) and an overall response rate of 45.0% (108/240); the completion rate among consenting respondents was 93.1% (108/116). Because part of the sample was recruited by snowball referral, the invitation denominator is approximate, and these rates should be read as estimates. No data were available on non-responders, so non-response bias cannot be quantified. The a priori power calculation for the primary multivariable logistic regression model (anticipated effect size OR ≈ 1.7 per +1 SD of literacy, two-sided α = 0.05, power = 0.80, event rate ≈ 0.45, 7 predictor parameters) indicated a minimum of 90 participants. At the anticipated event rate of approximately 0.45, a sample of 90 participants yields about 40–41 events, corresponding to an anticipated events-per-variable (EPV) ratio of roughly 5.8 for a seven-parameter model; the power calculation was therefore driven by the target odds ratio and α/power specification rather than by an EPV-of-10 rule. Reaching a conventional EPV of 10 would instead have required approximately 156 participants for seven parameters, and about 200 for the nine parameters ultimately estimated. The model actually fitted, however, estimated 9 predictor parameters against 52 events, an effective events-per-variable ratio of 5.8, which falls below the conventional threshold of 10 [24] and below the requirement implied by the sample-size criteria of Riley et al. [25]. The primary model is therefore susceptible to optimism and overfitting; this was addressed analytically through penalized estimation and bootstrap internal validation (Section 2.4) and is discussed as a limitation (Section 4.2). Convenience sampling is a limitation of this design and was addressed analytically through stratified subgroup reporting.

### 2.3. Measures and Survey Instruments

#### 2.3.1. The AI Economic Literacy Index (AIELI)

The AI Economic Literacy Index (AIELI) was developed for this study as a 25-item objective knowledge test (score 0–25) covering five conceptual domains: (i) cost-effectiveness analysis fundamentals (ICER, QALY, cost-per-life-year-gained); (ii) budget impact analysis and short-term affordability; (iii) Romanian HTA processes governed by ANMDMR; (iv) reimbursement mechanisms relevant to AI software-as-a-service products and DRG-coded oncology care; and (v) value-based pricing and risk-sharing agreements. Five items per domain were balanced between Romanian-specific operational knowledge and general health-economic principles, and each item was scored as correct (1) or incorrect (0). The instrument was developed de novo rather than adapted from an existing validated health-economics literacy measure; its item-generation, expert-review, cognitive-pretesting, and preliminary validation procedures are described in the following paragraphs.

The AIELI was developed de novo for this study. A structured search of the health-economics, HTA, and digital-health literature identified no existing validated instrument measuring objective knowledge of the economic and reimbursement concepts specific to clinical AI. Established digital-readiness and eHealth-literacy instruments, such as the eHealth Literacy Scale and the Digital Health Literacy Instrument, assess self-reported skills in locating and appraising digital health information rather than economic knowledge, and general pharmacoeconomic assessments do not address AI-specific pricing, procurement, or coding constructs [20,21]. Item development followed established scale-development guidance [26] and proceeded in four steps. First, a content blueprint of five conceptual domains was defined a priori from HTA and pharmacoeconomic reference sources and from the reimbursement literature on clinical AI [6,7,12,13]. Second, an initial pool of 34 single-best-answer items (six to eight per domain) was drafted by two authors with formal pharmacoeconomic training. Third, a panel of six external experts (two health economists, two medical oncologists, one hospital pharmacist, and one HTA analyst) independently rated every item for relevance and clarity on a four-point scale; item-level content validity indices (I-CVI) were computed, the 7 items with I-CVI < 0.78 were discarded, and the remaining 27 items showed a scale-level content validity index (average method) of S-CVI/Ave = 0.91. Fourth, cognitive interviews using concurrent think-aloud and retrospective verbal probing were conducted with eight oncology professionals to detect ambiguous stems, implausible distractors, and unintended cueing, following recommended practice for pretesting surveys in medical education and health services research [27]; the wording of nine items was revised on this basis.

The revised 27-item version was then piloted in the 14 oncology professionals described in Section 2.1, none of whom contributed to the final analytic sample. Pilot data were used to examine item difficulty (proportion answering correctly), corrected item-total correlations, and completion burden. Two items with negligible corrected item-total correlations (r < 0.10) and near-ceiling difficulty were removed, yielding the final 25-item instrument with 5 items per domain. In the analytic sample (*N* = 108), item difficulty ranged from 0.21 to 0.79 (mean 0.55), corrected item-total correlations ranged from 0.19 to 0.57 (median 0.34), and internal consistency was acceptable for a heterogeneous knowledge test (KR-20 = 0.79; 95% CI 0.72–0.85). No item, if deleted, raised KR-20 by more than 0.01.

With respect to conceptual structure, the AIELI was designed, scored, and analyzed as a unidimensional measure of AI-specific health-economic literacy. The five domains constitute a content-sampling blueprint whose purpose is to ensure adequate coverage of the construct; they were never intended to function as separately interpretable subscales, no domain subscores are reported, and a single total score (0–25) is used throughout. Consistent with that intention, a principal-component analysis of the 25 dichotomous items based on tetrachoric correlations yielded a dominant first component (eigenvalue 5.4; 21.7% of total variance) with a first-to-second eigenvalue ratio of 3.2, and all items loaded positively (0.21–0.63) on that component. These analyses are descriptive only. With 108 respondents and 25 dichotomous items, the sample is insufficient for confirmatory factor analysis or item-response-theory calibration, and the dimensional structure of the AIELI must be regarded as provisional pending replication in a larger independent sample. The participant-level k-means clustering reported in Section 3.7 partitions respondents, not items, and therefore provides no information about the dimensionality or psychometric validity of the AIELI.

#### 2.3.2. Attitudinal and Contextual Measures

**Figure 1:**
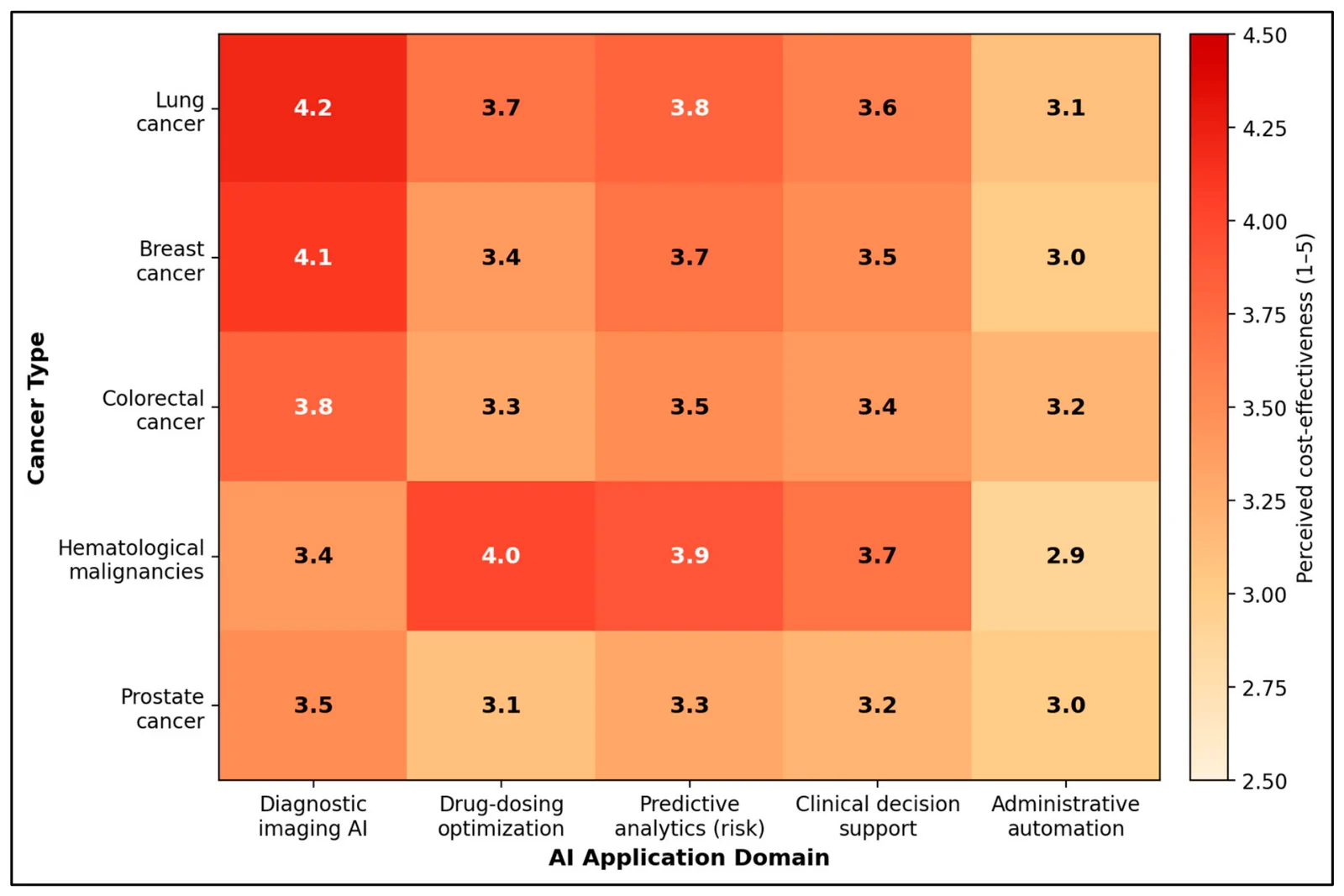
Heatmap of perceived cost-effectiveness of five AI application domains across five oncology subspecialties (mean 1–5 Likert scores; *N* = 108). Values are mean self-reported perceptions of cost-effectiveness and do not represent measured or modeled cost-effectiveness.

Continuous attitudinal constructs were measured on 1–5 Likert scales: ROI confidence (single item assessing self-perceived ability to estimate the return-on-investment of an AI tool in one’s own practice); willingness to invest (single item assessing stated support for, or willingness to recommend, allocating capital or recurring budget to AI in oncology over the next 12 months; because many respondents do not personally hold procurement or budgetary authority, this item is interpreted throughout as stated support for investment rather than as an enacted organizational investment decision); perceived financial barriers (4-item subscale: acquisition cost, recurring fees, integration costs, opportunity cost; mean score); cost-effectiveness perception (single item endorsing general cost-effectiveness of AI in oncology); and AI adoption intention (single item assessing near-term intention to use or support AI in routine oncology workflows). Reimbursement readiness was measured with a single 1–5 Likert item (“How ready do you consider the current reimbursement and payment system to be for funding AI tools in oncology in your setting?”; 1 = not at all ready, 5 = fully ready), scored so that higher values indicate greater perceived readiness of the payment infrastructure. It was treated as a single-item indicator and, like the other single-item measures, does not permit estimation of internal-consistency reliability; no multi-item reimbursement-readiness subscale was administered. Three additional contextual items captured prior AI training (yes/no), prior pharmacoeconomic training (yes/no), and current AI use in clinical or administrative practice (yes/no). Cancer-specific perceptions of cost-effectiveness were assessed across five oncology subspecialties and five AI application domains (Figure 1) using 1–5 ratings. The survey was administered electronically via a secure platform; median completion time was 16 min.

Four constructs used in the primary analyses—ROI confidence, willingness to invest, cost-effectiveness perception, and AI adoption intention—were assessed with single Likert items. Single-item measurement was adopted for three reasons. Each of these constructs is doubly concrete, in that the object (a specific AI tool in the respondent’s own oncology practice) and the attribute (confidence, endorsement, perceived cost-effectiveness, intention) are singular and unambiguous, a condition under which single items have repeatedly been shown to achieve predictive validity comparable to multi-item scales [28,29,30]. Response burden was the principal constraint identified during piloting, and the instrument had to be completable within a clinical working day. Finally, no validated multi-item Romanian-language scale exists for AI-specific ROI confidence or investment willingness. Perceived financial barriers, by contrast, were captured with a four-item subscale (acquisition cost, recurring fees, integration costs, opportunity cost; Cronbach’s α = 0.81) and analyzed as a mean score. We nevertheless emphasize the cost of this choice: single-item indicators do not permit estimation of internal-consistency reliability or of measurement error, attenuation due to unreliability cannot be quantified, and construct validity rests on content and face considerations rather than on empirical evidence of convergent and discriminant validity. This is treated as a principal limitation (Section 4.2), and multi-item validated scales should be developed and used for these constructs in future work.

### 2.4. Outcomes and Statistical Analysis

The primary outcome was willingness to invest in AI in oncology, analyzed both as a continuous variable (1–5) and dichotomized as high willingness to invest (score ≥ 4). The ≥4 cut point was prespecified. It reflects the operational question of interest—whether a respondent endorses, rather than merely tolerates, the allocation of capital or recurring budget to AI—and corresponds to the conventional “agree/strongly agree” boundary on a 1–5 endorsement scale. We recognize that dichotomizing an ordinal outcome discards information, reduces statistical power, and can distort effect estimates [31]. The dichotomized model is therefore reported as the primary analysis on grounds of interpretability, and two prespecified sensitivity analyses that retain the full ordinal information are reported alongside it: an ordinal (proportional-odds) logistic regression of the untransformed 1–5 response, and a linear regression treating willingness to invest as continuous. The proportional-odds assumption for the ordinal model was assessed with the Brant test (global χ^2^_9_ = 11.4, *p* = 0.25) and by inspection of parallel-slopes plots and was not violated. Secondary outcomes included AI adoption intention, ROI confidence, perceived financial barriers, and AIELI score. For profile discovery, we standardized AIELI, ROI confidence, willingness to invest, cost-effectiveness perception, adoption intention, and perceived financial barriers to z-scores prior to clustering. Missing data were limited. After exclusion of the three submissions with more than 25% missing responses, item-level missingness among the retained 108 respondents was low: no variable used in the primary analyses exceeded 4.6% missing, and missingness was 0% for willingness to invest (an inclusion criterion), 1.9% for AIELI total score, 2.8% for ROI confidence, 3.7% for reimbursement readiness, 4.6% for perceived financial barriers, and ≤3.7% for the remaining single-item measures. The logistic regression, mediation, and moderated-mediation models were fitted on complete cases for the variables in each model (*n* = 108 for the primary logistic model, since all its variables were complete after listwise handling of the few missing covariate values); the k-means clustering and the psychometric (KR-20, item-total) calculations were computed on respondents with complete data on the relevant variables; and the repeated-measures analysis of the cancer-type × AI-application grid used listwise-complete cases as described in Section 3.10. Given the low overall missingness, no imputation was performed, and complete-case and available-case results did not differ materially. Continuous variables are reported as mean ± SD (or median [IQR] when non-normal), and categorical variables as n (%). Between-group comparisons used independent-samples *t*-tests or one-way ANOVA for normally distributed continuous variables, Mann–Whitney U or Kruskal–Wallis tests for skewed distributions, and chi-square (or Fisher exact when expected counts < 5) for categorical variables. Bivariate associations were quantified using Spearman rank correlations (ρ) given the ordinal nature of the Likert items.

Multivariable logistic regression was used to identify factors independently associated with high willingness to invest. The complete and final adjustment set comprised five economic and capability predictors (AIELI per +1 SD; ROI confidence per +1 SD; perceived financial barriers per +1 SD; reimbursement readiness per +1 SD; prior AI training, yes vs. no) and four prespecified covariates (age per 10 years; female sex; private sector vs. public; academic sector vs. public), giving nine estimated predictor parameters in addition to the intercept. All nine were entered simultaneously into a single model, and no variable selection of any kind was performed; the variables listed above are exactly the variables in the model. Professional role was not retained in the primary model because six role categories would have added five further parameters to an already parameter-dense model and because role was strongly associated with sector; a sensitivity model additionally adjusted for role altered neither the direction nor the significance of any economic predictor. Linearity in the logit for the continuous predictors was assessed using the Box–Tidwell procedure and was not violated; multicollinearity was screened with variance inflation factors (all VIF < 2.0). Proportional-odds assumptions pertain to the ordinal sensitivity model described above and not to the binary model reported in the Results. With 52 events and 9 estimated predictor parameters the effective events-per-variable ratio was 5.8, below the conventional threshold of 10 [24]. Two safeguards were therefore applied: coefficients were re-estimated using Firth’s penalized-likelihood correction [32], and apparent model performance was corrected for optimism by 500-repetition bootstrap internal validation. Penalized and unpenalized estimates were directionally identical. Because the effective events-per-variable ratio (5.8) falls below the conventional threshold of 10, the Firth penalized-likelihood estimates—which reduce small-sample bias—are presented as the primary results for the logistic model, with the maximum-likelihood estimates reported alongside them for comparison. Effect estimates from the primary model should nonetheless be interpreted as provisional. Profile identification used k-means clustering on the six standardized features; the number of clusters (k = 3) was selected by combining silhouette analysis (peak average silhouette = 0.42 at k = 3) and the elbow criterion on within-cluster sum of squares. Because k-means is sensitive to initialization, and because an average silhouette of 0.42 indicates only moderate separation [33], three stability analyses were prespecified: (i) 1000 restarts of the Hartigan–Wong algorithm from 100 random initial centroid sets, recording the proportion of runs converging on the modal partition; (ii) non-parametric bootstrap resampling (1000 resamples) with cluster-wise Jaccard similarities computed by the clusterboot procedure [34]; and (iii) an independent agglomerative hierarchical clustering (Ward’s D2 linkage on Euclidean distances) cut at k = 3 and compared with the k-means partition using the adjusted Rand index [35]. Because differences between clusters on the six variables used to construct them are mathematically entailed by the clustering algorithm, such differences are reported descriptively and are not interpreted as evidence that the clusters are valid. External validation was performed against variables not used in clustering—professional role, sector, prior AI training, prior pharmacoeconomic training, and perceived reimbursement readiness—using chi-square and Kruskal–Wallis tests. Mediation analysis followed Hayes’ PROCESS macro Model 4 (Literacy → ROI confidence → willingness to invest), and moderated mediation followed PROCESS Model 7 with sector as the first-stage moderator, using 5000 bootstrap resamples for percentile confidence intervals. Both models adjusted for age, sex, professional role, and prior AI training; sector entered as the first-stage moderator through two dummy indicators with public hospital as the reference category. The index of moderated mediation was reported with its bootstrap CI. Subgroup sizes for the moderated-mediation model were 54 (public), 31 (private), and 23 (academic); with 23 respondents in the smallest stratum, the conditional indirect estimates are imprecise, and their bootstrap intervals should be read as descriptive rather than confirmatory. These are association models: they estimate whether the data are consistent with an indirect pathway and cannot establish temporal ordering or causal mechanism from cross-sectionally measured variables. All statistical tests were two-tailed with α = 0.05. Analyses were performed in IBM SPSS Statistics version 29.0 (IBM Corp., Armonk, NY, USA) and R version 4.4.1 (R Foundation for Statistical Computing, Vienna, Austria) using the psych, mediation, and cluster packages.

## 3. Results

### 3.1. Participant Characteristics

The final analytic sample comprised 108 oncology professionals with a mean age of 41.3 ± 10.7 years and a median of 12 years [IQR 7–19] of professional experience (Table 1). Women represented 58.3% (*n* = 63) of respondents, consistent with the gender composition of regional oncology services. The sectoral distribution was dominated by public hospitals (54/108, 50.0%), followed by private clinics (31/108, 28.7%) and academic settings (23/108, 21.3%); subgroup sizes for the sector-stratified models were therefore 54, 31, and 23, respectively. The professional mix was deliberately heterogeneous to support this study’s cross-functional framing: medical oncologists (28/108, 25.9%) and oncology nurses (24/108, 22.2%) formed the two largest clinical groups, while radiation oncologists contributed 15.7% (17/108) and hospital pharmacists 13.0% (14/108). Governance-facing professionals included administrators/managers responsible for oncology cost centers (13/108, 12.0%) and health economists with HTA or pharmacoeconomic experience (12/108, 11.1%); although these subgroups are smaller than the clinical groups, they were specifically over-represented relative to their general workforce share to enable role-level economic-reasoning comparisons. Prior AI-related training was reported by only 31.5% (34/108), while a higher proportion (43.5%, 47/108) reported prior pharmacoeconomic training. Current AI use in practice was reported by 38.0% (41/108).

### 3.2. Economic Constructs by Professional Role

Scores on the four core economic constructs differed across professional roles (Table 2), with health economists recording the highest AIELI score (19.4 ± 2.8/25) and the highest ROI confidence (3.7 ± 0.6/5), followed by administrators/managers (16.3 ± 3.5; 3.4 ± 0.7) and hospital pharmacists (15.7 ± 3.8; 3.2 ± 0.8). Among the clinical groups, medical oncologists recorded the highest values (AIELI 14.2 ± 4.3; ROI confidence 3.0 ± 0.8), while oncology nurses recorded the lowest scores across all four measures (AIELI 11.3 ± 4.4; ROI confidence 2.6 ± 0.9; willingness to invest 3.0 ± 0.9). Between-group differences were statistically significant for AIELI (one-way ANOVA F_5,102_ = 8.74, *p* < 0.001), ROI confidence (Kruskal–Wallis H = 18.93, *p* = 0.002), and willingness to invest (Kruskal–Wallis H = 11.62, *p* = 0.041) but not for adoption intention (*p* = 0.214). The highest-literacy group (health economists) did not record the highest willingness to invest (3.5 ± 0.7) or the highest adoption intention (3.4 ± 0.7), whereas oncology nurses recorded both the lowest AIELI and the lowest willingness to invest. Interpretation of these patterns is reserved for the Discussion.

### 3.3. Economic Constructs by Healthcare Sector

Sectoral comparison (Table 3) showed differences across the three financing environments. AIELI did not differ significantly across sectors (12.9 ± 4.5 in public hospitals, 14.7 ± 4.3 in private clinics, 14.3 ± 4.8 in academic centers; ANOVA *p* = 0.114). In contrast, ROI confidence was significantly higher in private clinics (3.2 ± 0.8) than in public hospitals (2.7 ± 0.8; ANOVA F_2,105_ = 3.84, *p* = 0.023), and willingness to invest showed an even larger gradient (3.7 ± 0.8 vs. 3.1 ± 0.9; F_2,105_ = 5.04, *p* = 0.008). Conversely, perceived financial barriers were highest in public hospitals (4.1 ± 0.7) and lowest in private clinics (3.6 ± 0.8; *p* = 0.012), and perceived reimbursement readiness mirrored this pattern (public 2.4 ± 0.8 vs. private 2.9 ± 0.9; *p* = 0.033). General cost-effectiveness perception and adoption intention did not differ significantly across sectors (*p* = 0.291 and *p* = 0.187, respectively).

### 3.4. Familiarity with Health Economic Concepts and AI Reimbursement

Self-reported familiarity with health economic concepts and AI-specific reimbursement structures (Table 4) revealed a pronounced gradient between general pharmacoeconomic vocabulary and AI-tailored reimbursement knowledge. The most familiar concept was cost-effectiveness analysis using ICER and QALY metrics (64/108, 59.3%), followed by Romanian DRG-coded oncology reimbursement (53/108, 49.1%), budget impact analysis (47/108, 43.5%), and the Romanian HTA process administered by ANMDMR (39/108, 36.1%). These results are consistent with the cohort’s relatively high rate of prior pharmacoeconomic training (43.5%) and the embeddedness of CEA/DRG vocabulary in routine oncology practice. By contrast, AI-specific economic concepts were considerably less familiar: value-based pricing and risk-sharing agreements (26/108, 24.1%), SaaS pricing models for clinical AI (22/108, 20.4%), the cost implications of EU AI Act high-risk classification (19/108, 17.6%), and AI-specific reimbursement codes from international precedent (18/108, 16.7%) were each endorsed by fewer than one in four respondents. The chi-square test of differential familiarity between traditional health-economic concepts (mean familiarity 47.0%) and AI-specific reimbursement concepts (mean familiarity 19.7%) yielded χ^2^_1_ = 67.3, *p* < 0.001.

### 3.5. Correlation Structure Among Economic Constructs

Bivariate Spearman correlations among the economic constructs are shown in Table 5. These unadjusted associations describe the correlation structure of the sample; by themselves, they do not establish the construct validity of the AIELI or of the attitudinal items. The strongest observed association was between AIELI and ROI confidence (ρ = 0.49, *p* < 0.001), so that objective economic knowledge and self-perceived ability to estimate the return-on-investment of AI tools were positively associated; this association motivated the mediation model examined below. ROI confidence was also correlated with willingness to invest (ρ = 0.43, *p* < 0.001), and AIELI showed a direct positive correlation with willingness to invest (ρ = 0.37, *p* < 0.001). Perceived reimbursement readiness was correlated with willingness to invest (ρ = 0.42, *p* < 0.001), comparable in magnitude to ROI confidence. The inverse correlation between perceived financial barriers and willingness to invest (ρ = −0.34, *p* < 0.001) was consistent with affordability concerns accompanying lower investment intentions, and a parallel but weaker inverse association was observed with adoption intention (ρ = −0.23, *p* = 0.017). Cost-effectiveness perception was associated with adoption intention (ρ = 0.38, *p* < 0.001) and, more modestly, with AIELI (ρ = 0.28, *p* = 0.003). Interpretation of this pattern is reserved for the Discussion.

### 3.6. Multivariable Correlates of High Willingness to Invest

After adjustment for demographic and structural covariates, four economic and capability factors remained independently associated with high willingness to invest (Table 6). Each one-SD increase in AIELI was associated with 78% higher odds of high willingness to invest (aOR 1.78, 95% CI 1.17–2.71, *p* = 0.007), and each one-SD increase in ROI confidence was associated with 93% higher odds (aOR 1.93, 95% CI 1.24–3.02, *p* = 0.004). The two coefficients were of similar magnitude; because all variables were measured at a single time point, their relative size carries no information about temporal ordering. Perceived financial barriers were inversely associated with high willingness to invest (aOR 0.58 per +1 SD, 95% CI 0.37–0.91, *p* = 0.018), and reimbursement readiness was associated with higher odds of investment (aOR 1.54 per +1 SD, 95% CI 1.01–2.36, *p* = 0.046); perceived payment infrastructure therefore remained associated with the outcome after adjustment for objective literacy. Respondents reporting prior AI training had approximately twice the adjusted odds of high willingness to invest (aOR 2.34, 95% CI 1.13–4.86, *p* = 0.022); the design does not permit the inference that training produced this difference. Private sector affiliation showed a borderline positive association (aOR 1.86, *p* = 0.074) that did not reach conventional significance after adjustment, while academic affiliation did not differ from public hospitals (aOR 1.23, *p* = 0.643). Demographic variables—age (*p* = 0.692) and female sex (*p* = 0.931)—were not significant. The model estimated 9 predictor parameters from 52 events, an effective events-per-variable ratio of 5.8. Apparent discrimination (C-statistic = 0.81) and calibration (Hosmer–Lemeshow χ^2^_8_ = 6.84, *p* = 0.554) are therefore likely to be optimistic. After 500-repetition bootstrap internal validation, the optimism-corrected C-statistic was 0.76 and the calibration slope 0.84, and Firth penalized-likelihood estimates were directionally identical with slightly attenuated odds ratios. The odds ratios above should accordingly be read as provisional and as susceptible to overfitting given the modest number of events. It should also be emphasized that the outcome modeled here is stated support for investment—that is, willingness to endorse or recommend allocating budget to oncology AI—rather than an enacted procurement or budget-allocation decision, because many respondents (including nurses and clinicians) do not personally hold formal capital or recurring-budget authority. The associations in Table 6 therefore describe correlates of expressed investment support, not of realized institutional spending.

### 3.7. Data-Driven Economic Adoption Profiles

K-means clustering on the six standardized economic features (AIELI, ROI confidence, cost-effectiveness perception, willingness to invest, adoption intention, perceived financial barriers) yielded a three-cluster solution with only moderate separation (average silhouette = 0.42; total within-cluster variance reduction = 58.4%) (Table 7). Because those same six variables were used to construct the partition, the between-cluster differences reported for them (rows 1–6 of Table 7; all *p* < 0.001) are mathematically expected and are presented descriptively; they are not independent evidence that the clusters are meaningful. The cluster labels below are descriptive shorthand for the observed centroid patterns rather than externally validated phenotypes. Cluster 1, labeled “Cost-Conscious Adopters” (*n* = 43, 39.8%), combined the highest AIELI (15.6 ± 3.7), highest ROI confidence (3.4 ± 0.7), highest willingness to invest (3.9 ± 0.7), and highest adoption intention (4.1 ± 0.6) with the lowest perceived financial barriers (3.3 ± 0.7) and substantially elevated reimbursement readiness (3.1 ± 0.8); 48.8% had prior AI training and 41.9% worked in private clinics. Cluster 2, “Reimbursement-Cautious” (*n* = 37, 34.3%), occupied an intermediate literacy and ROI position but exhibited high perceived financial barriers (4.2 ± 0.6) and low reimbursement readiness (2.4 ± 0.7). Cluster 3, “Budget-Constrained Skeptics” (*n* = 28, 25.9%), showed the lowest scores on every favorable construct (AIELI 10.7 ± 4.1; ROI confidence 2.3 ± 0.7; willingness 2.6 ± 0.8; adoption 2.9 ± 0.8) alongside the highest perceived barriers (4.3 ± 0.7). Three variables that were not used to generate the partition differed across clusters: prior AI training (χ^2^_2_ = 11.49, *p* = 0.003), private-sector membership (χ^2^_2_ = 7.28, *p* = 0.026), and perceived reimbursement readiness (*p* < 0.001; Table 7). Professional role also differed across clusters (χ^2^_10_ = 21.4, *p* = 0.018), with health economists and administrators over-represented among Cost-Conscious Adopters and oncology nurses over-represented among Budget-Constrained Skeptics. These external associations offer limited convergent support for the partition but do not establish that the three groups are stable phenotypes. Stability analyses were correspondingly mixed. The modal partition was recovered in 82.6% of 1000 random restarts. Cluster-wise bootstrap Jaccard similarities were 0.79 for Cost-Conscious Adopters, 0.61 for Reimbursement-Cautious, and 0.72 for Budget-Constrained Skeptics; only the first exceeded the conventional 0.75 threshold for a stable cluster, with values between 0.60 and 0.75 indicating a pattern that is suggestive rather than reliable [34]. Agglomerative hierarchical clustering (Ward’s D2) cut at k = 3 agreed only moderately with the k-means partition (adjusted Rand index 0.58). The three-cluster solution is therefore treated as exploratory throughout. The pattern of centroids, moreover, indicates that the three clusters largely trace a single continuum running from more favorable to less favorable responses across several positively correlated constructs—AIELI, ROI confidence, cost-effectiveness perception, willingness to invest, and adoption intention all decline together from Cluster 1 to Cluster 3, while perceived financial barriers rise in parallel—rather than identifying qualitatively distinct, crossing profiles. Combined with the moderate silhouette value, the mixed cluster-wise Jaccard stability, and the only moderate agreement with hierarchical clustering, this argues for interpreting the partition as a coarse gradient of economic favorability rather than as a set of discrete phenotypes.

### 3.8. Mediation Analysis: AIELI → ROI Confidence → Willingness to Invest

The covariate-adjusted mediation model (Table 8) was consistent with an indirect association between AI economic literacy and willingness to invest via ROI confidence. The first-stage path was estimated at β = 0.43 (SE = 0.08, *p* < 0.001) so that each one-unit increase in standardized AIELI was associated with a 0.43-unit increase in ROI confidence after adjustment for age, sex, sector, role, and prior AI training. The second-stage path was also significant (b: β = 0.31, SE = 0.09, *p* = 0.001), indicating that ROI confidence was associated with willingness to invest independently of objective literacy. The direct effect of AIELI on willingness to invest remained significant after introducing ROI confidence as a mediator (c′: β = 0.17, SE = 0.07, *p* = 0.018), and the total effect was β = 0.30 (*p* < 0.001), consistent with partial rather than full mediation. The bootstrapped indirect effect was 0.13 (95% CI [0.058, 0.231]); the interval excludes zero, which is consistent with an indirect association but does not confirm mediation in the causal sense, because all three variables were measured at the same time point. The proportion of the total association accounted for by the indirect path was 44.3%. That quantity is model-dependent and, in a cross-sectional design, describes a statistical decomposition rather than a mechanism: the reverse ordering, in which prior investment experience shapes ROI confidence and measured literacy alike, is equally compatible with these data.

### 3.9. Moderated Mediation: Sector as First-Stage Moderator

The moderated mediation model (Table 9) examined whether the strength of the literacy → ROI confidence association, and consequently the indirect association with willingness to invest, differed by sector. The AIELI × sector interaction was significant in the first-stage equation (*p* = 0.014), and the conditional first-stage coefficients varied across sectors: a_1_ = 0.23 (SE = 0.10, *p* = 0.024) in public hospitals; a_2_ = 0.45 (SE = 0.13, *p* < 0.001) in academic centers; and a_3_ = 0.62 (SE = 0.11, *p* < 0.001) in private clinics. Combined with the second-stage path (b: β = 0.31, *p* = 0.001), this produced sector-conditional indirect estimates of β = 0.072 [95% bootstrap CI 0.014, 0.158] in public hospitals, β = 0.141 [0.043, 0.267] in academic centers, and β = 0.193 [0.087, 0.318] in private clinics. The index of moderated mediation comparing private clinics with public hospitals was 0.061 [0.012, 0.131]; the bootstrap interval excludes zero, which is consistent with sector-conditional variation in the first-stage association. Descriptively, the estimated conditional indirect association in private clinics was approximately 2.7 times that in public hospitals. Because the subgroups are small (public *n* = 54, private *n* = 31, academic *n* = 23) and the bootstrap intervals overlap substantially, this ratio is imprecisely estimated and should not be treated as a stable effect size. Because sector is a three-level moderator, all pairwise contrasts are reported rather than the private-versus-public comparison alone. In the first-stage equation, the two moderator-by-literacy interaction terms (relative to the public-hospital reference) were 0.22 (SE = 0.13; 95% bootstrap CI −0.03 to 0.47) for the academic sector and 0.39 (SE = 0.12; 95% bootstrap CI 0.15 to 0.63) for private clinics, so only the private-versus-public interaction was clearly distinguishable from zero. The corresponding indices of moderated mediation were 0.061 (95% bootstrap CI 0.012 to 0.131) for private versus public, 0.034 (95% bootstrap CI −0.006 to 0.086) for academic versus public, and 0.027 (95% bootstrap CI −0.011 to 0.079) for private versus academic. The bootstrap intervals for the academic-versus-public and private-versus-academic indices include zero, so the only contrast for which the sector-conditional indirect association differed detectably was private versus public; the academic sector occupied an intermediate but statistically indistinguishable position. Structural interpretation of the sectoral pattern is deferred to the Discussion.

### 3.10. Cancer-Type and AI-Application Heterogeneity in Cost-Effectiveness Perception

Figure 1 illustrates marked heterogeneity in perceived cost-effectiveness across the cancer-type × AI-application matrix, with mean ratings ranging from 2.9 (administrative automation in hematological malignancies) to 4.2 (diagnostic imaging AI in lung cancer). Three patterns are evident. First, diagnostic imaging AI was the highest-rated application for lung cancer (4.2), breast cancer (4.1), and colorectal cancer (3.8), consistent with the relative maturity of imaging-AI evidence in these radiologically intensive cancers. Second, drug-dosing optimization and predictive analytics were rated highest in hematological malignancies (4.0 and 3.9, respectively), reflecting the complexity of dose individualization and risk stratification in leukemia/lymphoma care. Third, administrative automation received the lowest ratings across all five cancer types (range 2.9–3.2). A 5 × 5 repeated-measures ANOVA on within-respondent ratings showed significant main effects of cancer type (F_4,412_ = 14.6, *p* < 0.001) and AI application (F_4,412_ = 28.3, *p* < 0.001), and a significant interaction (F_16,1648_ = 6.7, *p* < 0.001), indicating that perceived cost-effectiveness varied jointly with both dimensions. Importantly, prostate cancer received uniformly modest ratings across all AI applications (range 3.0–3.5), an observation that may reflect the predominance of cost-effective biomarker-driven (PSA) workflows in this disease and a narrower perceived AI value-add. Because the two five-level within-subject factors can violate the sphericity assumption, Mauchly’s test was applied to each within-subject effect; sphericity was rejected for cancer type (W = 0.71, *p* = 0.008), for AI application (W = 0.68, *p* = 0.004), and for their interaction (W = 0.42, *p* < 0.001). All within-subject F-tests were therefore Greenhouse–Geisser corrected (ε = 0.86, 0.83 and 0.71 for the three effects, respectively), and Huynh–Feldt-corrected tests gave identical conclusions. The corrected tests remained significant for cancer type (*p* < 0.001), AI application (*p* < 0.001), and their interaction (*p* < 0.001). The degrees of freedom reported for the interaction are the uncorrected values (16, 1648), obtained as (5 − 1)(5 − 1) for the numerator and (5 − 1)(5 − 1)(104 − 1) for the error term. Effect sizes were moderate to large (partial η^2^ = 0.12 for cancer type, 0.21 for AI application, and 0.09 for the interaction). The cancer-type × AI-application ratings formed a complete 25-cell within-respondent grid, and the repeated-measures model requires a complete grid for every respondent; the four respondents with one or more missing cells were therefore excluded list-wise, so this analysis is based on the 104 respondents with complete rating matrices rather than on all 108.

### 3.11. Sector-Conditional Indirect Effects on Willingness to Invest

**Figure 2:**
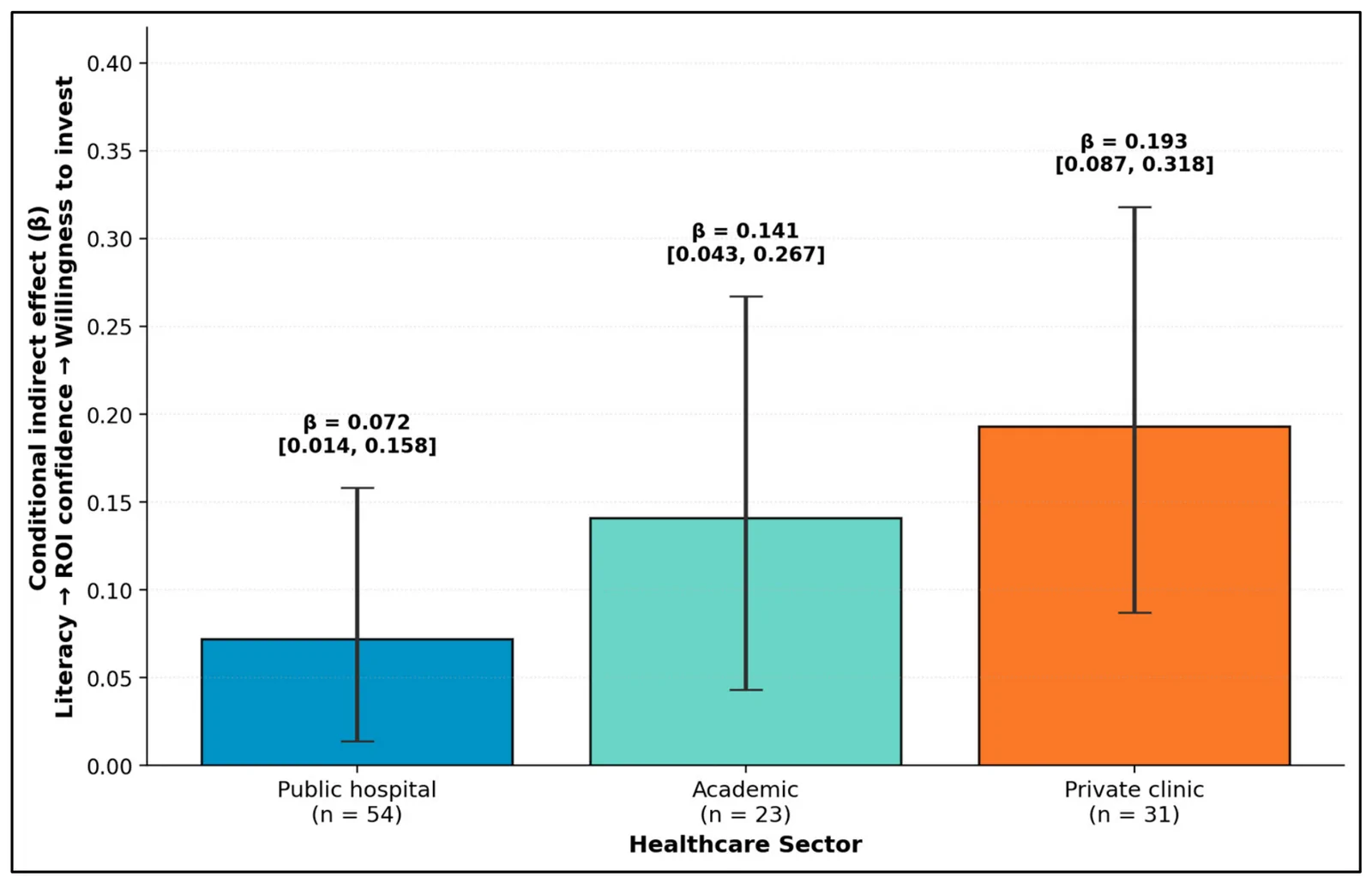
Sector-conditional indirect effects of AI economic literacy on willingness to invest, mediated by ROI confidence (bootstrap 95% confidence intervals; B = 5000). The plotted quantities are model-based conditional indirect associations estimated from cross-sectional data; they do not represent demonstrated causal pathways, and the differences between sectors are estimated from subgroups of 54, 31, and 23 respondents. Bar colors denote healthcare sector only (blue, public hospital; green, academic center; orange, private clinic) and carry no additional meaning; error bars are bootstrap 95% confidence intervals.

Figure 2 visualizes the magnitude of the literacy-to-investment indirect pathway across the three healthcare sectors, with the confidence interval for each conditional indirect effect derived from 5000 bootstrap resamples. The conditional indirect estimate in private clinics (β = 0.193, 95% CI [0.087, 0.318]) was approximately 2.7-fold larger than in public hospitals (β = 0.072, 95% CI [0.014, 0.158]), with academic centers occupying an intermediate position (β = 0.141, 95% CI [0.043, 0.267]). All three sector-specific intervals excluded zero, so each was consistent with a non-zero indirect association; the intervals nonetheless overlap substantially, and the apparent differences in magnitude are imprecisely estimated. The index of moderated mediation (β = 0.061, 95% CI [0.012, 0.131]) quantifies the difference between the private and public conditional indirect estimates, and its interval excludes zero.

## 4. Discussion

### 4.1. Analysis of Findings

This cross-sectional study of 108 Romanian oncology professionals characterized economic correlates of AI adoption intentions in a setting where, to our knowledge, they have not previously been described. The combination of a 25-item objective economic literacy index (mean AIELI 13.7 ± 4.6/25, moderate) with disaggregated attitudinal scales showed a coherent correlational structure: AI economic literacy was associated with ROI confidence (ρ = 0.49), which was in turn associated with willingness to invest (ρ = 0.43), while perceived financial barriers were independently and inversely associated with the outcome (aOR 0.58 per +1 SD). The familiarity asymmetry between traditional health-economic concepts and AI-specific reimbursement structures (e.g., 59.3% for CEA but only 16.7% for AI-specific reimbursement codes) may indicate that the cohort’s general pharmacoeconomic literacy has not yet extended to AI-specific operational competence—an observation consistent with the broader European observation that AI-economics literacy lags behind AI-clinical literacy and that this gap is particularly acute in budget-constrained systems [36,37].

A central observation of this study is that the strength of the literacy–confidence–investment association differed by sector. The conditional indirect effect was approximately 2.7-fold larger in private clinics (β = 0.193) than in public hospitals (β = 0.072), with academic centers in an intermediate position (β = 0.141), and the index of moderated mediation (0.061; 95% CI 0.012–0.131) excluded zero. One structural reading of this pattern is that in private clinics, where AI investment decisions are more directly responsive to professional input and revenue capture, higher literacy may more readily be accompanied by ROI confidence and by investment willingness, whereas in public hospitals, where AI procurement is gated by external CNAS budget allocations and slow national tariff revision, higher literacy may not be accompanied by investment intentions [38]. This is a plausible interpretation, not a demonstrated mechanism. A cross-sectional design cannot distinguish it from the alternative that professionals working in investment-permissive environments acquire economic literacy and confidence through repeated exposure to procurement decisions, nor from residual confounding by unmeasured institutional characteristics. The pattern parallels organizational–economic studies in other Eastern European healthcare systems and is consistent with the implementation-science principle that capability is necessary but not sufficient without enabling structural conditions [39]. Given subgroup sizes of 54, 31, and 23, it should not be generalized to the Romanian public, private, and academic oncology sectors as a whole.

The exploratory profiles (Cost-Conscious Adopters, Reimbursement-Cautious, Budget-Constrained Skeptics) describe candidate subgroups within this dataset. The “Reimbursement-Cautious” profile is of particular interest because it combines moderate literacy (AIELI 13.4 ± 3.9) with the highest perceived financial barriers (4.2 ± 0.6) and low reimbursement readiness (2.4 ± 0.7), a configuration consistent with economic understanding failing to produce economic optimism where the underlying payment infrastructure is perceived as unfavorable. This profile resonates with international evidence that AI-specific reimbursement gaps suppress investment even among informed professionals [19,40]. Whether targeting this group with reimbursement-clarity interventions—practical templates for value-based contracting, simulated DRG-bundling scenarios, and exposure to international AI-coding precedents—would increase adoption is a hypothesis for future intervention studies; the present data cannot answer it. The same applies to the tentative suggestions that Cost-Conscious Adopters might be the group for which scale-up is most feasible, and that Budget-Constrained Skeptics might need foundational literacy and confidence-building before reimbursement-specific measures could take effect. These groupings were only moderately stable under resampling (cluster-wise Jaccard 0.61–0.79; adjusted Rand index 0.58 against hierarchical clustering) and are exploratory descriptions of this sample rather than validated phenotypes.

Because reimbursement readiness is foundational to the title and framework of this study, its practical meaning in the Romanian setting warrants concrete description. First, public oncology activity is contracted annually by the National Health Insurance House (CNAS) with individual hospitals under negotiated ceilings, and inpatient episodes are settled through a DRG system in which the tariff attaches to the case rather than to the diagnostic technology used to produce it. An AI tool that shortens reporting time or improves nodule detection therefore generates no incremental revenue for the hospital, while its cost falls on the same capped budget line that funds staff and consumables; because national DRG tariffs are revised infrequently, value created by an AI tool is absorbed rather than remunerated. This is the institutional counterpart of the low reimbursement-readiness ratings recorded in public hospitals (2.4 ± 0.8). Second, most clinical AI is licensed as software-as-a-service, so that costs are recurring—annual subscription, per-study fees, model revalidation, drift monitoring—rather than capital, whereas Romanian public procurement and hospital accounting remain organized around one-off capital equipment purchases with depreciation schedules. Recurring software costs must consequently be accommodated within operating budgets that are fixed before the procurement decision is taken. Third, the national HTA process administered by ANMDMR was designed around pharmaceuticals: dossiers are appraised against therapeutic-added-value criteria and against reimbursement decisions in a defined set of reference countries, and digital health technologies map onto that apparatus only awkwardly; no AI-specific tariff or coding pathway currently exists. Fourth, the compliance costs introduced by the EU Artificial Intelligence Act for high-risk medical AI—technical documentation, post-market monitoring, conformity assessment—are borne by manufacturers and, indirectly, by purchasers and are reflected in no existing tariff. Taken together, these four features describe an environment in which a professional may simultaneously and correctly judge an AI tool to be cost-effective at system level and unaffordable at the level of their own cost center. The observation that reimbursement readiness was independently associated with willingness to invest (aOR 1.54 per +1 SD) is consistent with that account, although it does not establish it.

Two constraints bound the interpretation of everything reported above. First, the sample is a convenience sample recruited by institutional invitation and snowball referral from six institutions in a single region of western Romania, with an estimated response rate of 45% and no information on non-responders. These findings describe this cohort. They should not be generalized to Romanian oncology professionals as a whole, and still less to Eastern European health systems more broadly, until they are replicated in larger, nationally representative samples. Second, and more fundamentally, this study identifies associations. It shows that professionals with higher AI economic literacy and higher ROI confidence also expressed greater willingness to invest, and that perceived financial barriers and reimbursement readiness were associated with that willingness in the expected directions. It does not show that raising literacy raises investment. The educational and policy measures suggested by these patterns—AI-specific health-economics modules within oncology training, worked value-based contracting templates, simulated DRG-bundling exercises, structured exposure to international AI coding precedents—should therefore be read as plausible and testable hypotheses for future intervention research rather than as conclusions established by the present data. A stepped-wedge or cluster-randomized evaluation, with willingness to invest measured before and after the intervention and with actual procurement or contracting decisions as a distal outcome, would be the appropriate next step.

### 4.2. Study Limitations

Several limitations should be acknowledged when interpreting these findings. First, the cross-sectional design precludes causal inference; while the moderated-mediation analysis is consistent with a plausible pathway linking literacy, confidence, sector, and willingness to invest, longitudinal or experimental designs would be required to confirm directionality and rule out reverse causation (e.g., that prior investment experience boosts ROI confidence rather than the reverse). Second, the use of convenience sampling at “Victor Babes” University of Medicine and Pharmacy Timisoara and affiliated oncology services means that findings may not generalize fully to other Romanian regions or to other Eastern European countries with different reimbursement infrastructures, although the over-sampling of governance-facing roles strengthens within-study comparisons. Third, attitudinal constructs (ROI confidence, reimbursement readiness, perceived financial barriers, willingness to invest) were self-reported and may be influenced by social desirability or by recent organizational events; objective behavioral outcomes such as actual procurement decisions or contract negotiations were not measured. Relatedly, we did not record whether individual respondents held formal procurement or budgetary authority, and many participants—particularly oncology nurses and clinicians—are unlikely to control capital or recurring institutional budgets. The primary outcome should therefore be understood as stated support for, or willingness to recommend, investment rather than as an actual organizational investment decision, and the absence of information on respondents’ procurement or budgetary authority is itself a limitation that constrains the interpretation of the willingness-to-invest measure. Fourth, the AIELI, while internally consistent (KR-20 = 0.79), is a novel instrument that requires external validation in additional populations before being used as a benchmarking tool. Fifth, subgroup sizes for the smaller professional roles (health economists *n* = 12; administrators *n* = 13) limit precision for role-level comparisons and may inflate the standard errors of role-specific estimates. Sixth, our cancer-by-AI-application matrix relies on retrospective perception rather than actual implementation outcomes and may be influenced by media exposure rather than direct evidence. Seventh, the dichotomization of willingness to invest at ≥4 was prespecified but inevitably loses information and statistical power; prespecified sensitivity analyses using ordinal (proportional-odds) and linear models of the untransformed outcome yielded directionally consistent results.

Several further limitations warrant explicit statement. Four of this study’s core constructs—ROI confidence, willingness to invest, cost-effectiveness perception, and adoption intention—were measured with single Likert items, which precludes estimation of internal-consistency reliability and of measurement error and leaves construct validity resting on content and face considerations; associations involving these variables may be attenuated by unreliability to an unknown degree. Because predictors and outcomes were collected from the same respondents, through the same instrument, at the same sitting, common-method variance may have inflated the observed associations [41]; procedural remedies (anonymity, separation of the knowledge test from the attitudinal block, mixed item polarity) were applied, but no marker variable was included and no statistical correction was therefore possible. Willingness to invest and adoption intention are also socially desirable positions in a professional environment in which AI is widely promoted, and self-reports may exceed what respondents would endorse in a budget meeting. The primary logistic model estimated 9 parameters from 52 events, an events-per-variable ratio of 5.8, so the reported odds ratios remain liable to optimism even after penalization and internal validation; and the moderated-mediation model rests on subgroups of 54, 31, and 23 respondents, which renders the conditional indirect estimates sensitive to sampling variability. The k-means solution was only moderately stable under resampling (cluster-wise Jaccard 0.61–0.79) and only moderately concordant with hierarchical clustering (adjusted Rand index 0.58); the three groups are exploratory descriptions of this dataset rather than validated phenotypes, and the between-cluster differences on the six clustering variables are mathematically entailed by the clustering procedure rather than constituting independent evidence of group structure. Respondents were clustered within six institutions, and no multilevel adjustment was made, so standard errors may be understated. Finally, the AIELI is a new instrument whose dimensional structure could not be formally tested at this sample size; the psychometric evidence presented in Section 2.3.1 is preliminary and requires confirmation in an independent sample.

## 5. Conclusions

In this exploratory cross-sectional sample of Romanian oncology professionals, AI adoption intentions and willingness to invest were associated with three potentially modifiable economic factors—objective economic literacy, ROI confidence, and perceived financial barriers—with reimbursement readiness independently associated with the outcome. The strength of the literacy-to-investment association differed by sector, the conditional indirect estimate in private clinics being approximately 2.7-fold that in public hospitals. This may suggest that capability-building interventions would need to be accompanied by structural reimbursement reform in budget-fixed environments, a proposition that requires prospective testing rather than one established here. Three exploratory economic profiles—Cost-Conscious Adopters, Reimbursement-Cautious, and Budget-Constrained Skeptics—offer a provisional stratification. Because this clustering solution was only internally and exploratorily validated and appears to trace a favorable-to-unfavorable continuum rather than discrete phenotypes, we do not prescribe profile-specific interventions here. The possibilities that foundational literacy and confidence training might suit lower-scoring respondents, that reimbursement-clarity and value-based-contracting support might suit those with high perceived barriers, and that scale-up and governance support might suit higher-scoring respondents are offered only as hypotheses requiring external validation in independent samples and prospective evaluation, not as recommendations established by these data. The pronounced familiarity asymmetry between traditional pharmacoeconomic concepts and AI-specific reimbursement structures may indicate a training priority. Perceived cost-effectiveness varied by cancer type and AI application, with imaging AI in lung and breast cancers and predictive analytics in hematological malignancies rated most favorably; such perceptions may inform, but cannot substitute for, formal economic evaluation in AI capital budgeting. Whether policy interventions integrating literacy, reimbursement infrastructure, and profile-stratified training outperform uniform top-down deployment is a hypothesis generated by, and not established by, this cross-sectional study.

## Figures and Tables

**Table 1:** Participant characteristics (*N* = 108).

Characteristic	Value
Age, years (mean ± SD)	41.3 ± 10.7
Years of professional experience, median [IQR]	12 [7,8,9,10,11,12,13,14,15,16,17,18,19]
Female sex, n (%)	63 (58.3%)
Sector: public hospital	54 (50.0%)
Sector: private clinic	31 (28.7%)
Sector: academic/university	23 (21.3%)
Profession: medical oncologists	28 (25.9%)
Profession: radiation oncologists	17 (15.7%)
Profession: oncology nurses	24 (22.2%)
Profession: hospital pharmacists	14 (13.0%)
Profession: administrators/managers	13 (12.0%)
Profession: health economists	12 (11.1%)
Prior AI-related training (any), n (%)	34 (31.5%)
Prior pharmacoeconomic training, n (%)	47 (43.5%)
Current AI use in practice, n (%)	41 (38.0%)

**Table 2:** Key economic constructs by professional role (mean ± SD).

Professional Role	*n*	AIELI (0–25)	ROI Confidence (1–5)	Willingness to Invest (1–5)	Adoption Intention (1–5)
Medical oncologists	28	14.2 ± 4.3	3.0 ± 0.8	3.6 ± 0.8	3.8 ± 0.7
Radiation oncologists	17	13.8 ± 4.1	2.8 ± 0.7	3.4 ± 0.9	3.7 ± 0.7
Oncology nurses	24	11.3 ± 4.4	2.6 ± 0.9	3.0 ± 0.9	3.4 ± 0.8
Hospital pharmacists	14	15.7 ± 3.8	3.2 ± 0.8	3.4 ± 0.8	3.5 ± 0.8
Administrators/Managers	13	16.3 ± 3.5	3.4 ± 0.7	3.6 ± 0.7	3.7 ± 0.6
Health economists	12	19.4 ± 2.8	3.7 ± 0.6	3.5 ± 0.7	3.4 ± 0.7
Overall (*N* = 108)	108	13.7 ± 4.6	2.9 ± 0.8	3.3 ± 0.9	3.6 ± 0.8
*p*-value (ANOVA/Kruskal–Wallis)	—	<0.001	0.002	0.041	0.214

**Table 3:** Economic constructs by healthcare sector (mean ± SD).

Construct	Public Hospital (*n* = 54)	Private Clinic (*n* = 31)	Academic (*n* = 23)	*p*-Value
AIELI (0–25)	12.9 ± 4.5	14.7 ± 4.3	14.3 ± 4.8	0.114
ROI confidence (1–5)	2.7 ± 0.8	3.2 ± 0.8	2.9 ± 0.9	0.023
Willingness to invest (1–5)	3.1 ± 0.9	3.7 ± 0.8	3.2 ± 0.9	0.008
Cost-effectiveness perception (1–5)	3.3 ± 0.9	3.6 ± 0.8	3.4 ± 0.9	0.291
Perceived financial barriers (1–5)	4.1 ± 0.7	3.6 ± 0.8	3.9 ± 0.8	0.012
Reimbursement readiness (1–5)	2.4 ± 0.8	2.9 ± 0.9	2.6 ± 0.9	0.033
Adoption intention (1–5)	3.5 ± 0.8	3.8 ± 0.7	3.6 ± 0.8	0.187

**Table 4:** Self-reported familiarity with health economic concepts and AI reimbursement structures. Familiarity was self-reported on a single dichotomous item per concept (“Are you familiar with this concept?”, yes/no); the second column reports the number and percentage of the 108 respondents answering yes.

Concept/Framework	Respondents Reporting Familiarity, *n* (%)
Cost-effectiveness analysis (ICER, QALY)	64 (59.3%)
Budget impact analysis (BIA)	47 (43.5%)
Romanian HTA process (ANMDMR)	39 (36.1%)
Romanian DRG-coded oncology reimbursement	53 (49.1%)
Value-based pricing and risk-sharing agreements	26 (24.1%)
Software-as-a-service (SaaS) pricing models for clinical AI	22 (20.4%)
EU AI Act—cost implications of high-risk classification	19 (17.6%)
AI-specific reimbursement codes (international precedent)	18 (16.7%)

Denominator is *N* = 108 for every row. Values are counts of respondents endorsing familiarity, with the corresponding row percentage in parentheses. Rows are not mutually exclusive. ANMDMR, Romanian Agency for Medicines and Medical Devices; BIA, budget impact analysis; DRG, diagnosis-related group; HTA, health technology assessment; ICER, incremental cost-effectiveness ratio; QALY, quality-adjusted life year; SaaS, software-as-a-service.

**Table 5:** Spearman correlations among key economic measures.

Variable Pair	Spearman’s ρ	*p*-Value
AIELI vs. ROI confidence	0.49	<0.001
ROI confidence vs. willingness to invest	0.43	<0.001
AIELI vs. willingness to invest	0.37	<0.001
Cost-effectiveness perception vs. adoption intention	0.38	<0.001
Financial barriers vs. willingness to invest	−0.34	<0.001
Reimbursement readiness vs. willingness to invest	0.42	<0.001
Budget impact awareness vs. adoption intention	0.31	0.001
AIELI vs. cost-effectiveness perception	0.28	0.003
Financial barriers vs. adoption intention	−0.23	0.017

ρ denotes Spearman’s rank-order correlation coefficient, reported for each pair of variables listed in the first column; a negative sign indicates an inverse association. All coefficients are unadjusted bivariate associations computed on the full analytic sample (*N* = 108); *p*-values are two-tailed and are not corrected for multiple comparisons. AIELI, AI Economic Literacy Index; ROI, return on investment.

**Table 6:** Multivariable logistic regression predicting high willingness to invest (≥4). All nine predictors listed below were entered simultaneously into a single model; the table reports the complete adjustment set. Because the effective events-per-variable ratio was below 10, the Firth penalized-likelihood adjusted odds ratios are presented as the primary estimates (second column), with the unpenalized maximum-likelihood estimates shown alongside them (third column) for comparison; the two sets of estimates are directionally identical, with the penalized odds ratios slightly attenuated toward the null. *p*-values correspond to the maximum-likelihood model.

Predictor	Firth Penalized aOR (95% CI)	ML aOR (95% CI)	*p*-Value
AIELI (per +1 SD)	1.68 (1.15–2.45)	1.78 (1.17–2.71)	0.007
ROI confidence (per +1 SD)	1.81 (1.21–2.70)	1.93 (1.24–3.02)	0.004
Perceived financial barriers (per +1 SD)	0.61 (0.41–0.92)	0.58 (0.37–0.91)	0.018
Reimbursement readiness (per +1 SD)	1.47 (1.01–2.17)	1.54 (1.01–2.36)	0.046
Prior AI training (yes vs. no)	2.15 (1.12–4.15)	2.34 (1.13–4.86)	0.022
Private sector (vs. public)	1.75 (0.95–3.23)	1.86 (0.94–3.68)	0.074
Academic sector (vs. public)	1.20 (0.55–2.66)	1.23 (0.51–2.97)	0.643
Age (per 10 years)	0.95 (0.72–1.25)	0.94 (0.69–1.28)	0.692
Female sex (vs. male)	0.97 (0.52–1.83)	0.97 (0.48–1.96)	0.931

Outcome: high willingness to invest (score ≥ 4 on a 1–5 scale). Adjustment set: AIELI, ROI confidence, perceived financial barriers, reimbursement readiness, prior AI training, sector (two dummy indicators, public hospital reference), age, and sex—9 estimated predictor parameters plus intercept. Events = 52; non-events = 56; effective events-per-variable ratio = 5.8. Apparent C-statistic 0.81; optimism-corrected C-statistic 0.76 and calibration slope 0.84 after 500-repetition bootstrap internal validation; Hosmer–Lemeshow χ^2^_8_ = 6.84, *p* = 0.554; all VIF < 2.0. Estimates are model-based associations from cross-sectional data and do not represent causal effects. aOR, adjusted odds ratio; CI, confidence interval; SD, standard deviation.

**Table 7:** Exploratory economic adoption profiles (k-means clustering on standardized features; ANOVA or chi-square across clusters).

Variable	Cost-Conscious Adopters (*n* = 43)	Reimbursement-Cautious (*n* = 37)	Budget-Constrained Skeptics (*n* = 28)	*p*-Value
AIELI (0–25)	15.6 ± 3.7	13.4 ± 3.9	10.7 ± 4.1	<0.001
ROI confidence (1–5)	3.4 ± 0.7	2.9 ± 0.7	2.3 ± 0.7	<0.001
Cost-effectiveness perception (1–5)	3.9 ± 0.6	3.3 ± 0.7	2.8 ± 0.8	<0.001
Willingness to invest (1–5)	3.9 ± 0.7	3.2 ± 0.8	2.6 ± 0.8	<0.001
Adoption intention (1–5)	4.1 ± 0.6	3.6 ± 0.7	2.9 ± 0.8	<0.001
Perceived financial barriers (1–5)	3.3 ± 0.7	4.2 ± 0.6	4.3 ± 0.7	<0.001
Reimbursement readiness (1–5)	3.1 ± 0.8	2.4 ± 0.7	2.1 ± 0.8	<0.001
Prior AI training, n (%)	21 (48.8%)	9 (24.3%)	4 (14.3%)	0.003
Private sector membership, n (%)	18 (41.9%)	9 (24.3%)	4 (14.3%)	0.026

Clustering variables (rows 1–6): AIELI, ROI confidence, cost-effectiveness perception, willingness to invest, adoption intention, and perceived financial barriers were the six standardized features used to generate the partition. Differences between clusters on these six variables are mathematically entailed by the clustering algorithm and are reported for description only; they are not evidence that the clusters are valid. External variables (rows 7–9): reimbursement readiness, prior AI training, and private-sector membership were not used in clustering and therefore provide independent comparisons. Average silhouette = 0.42 (moderate separation). Cluster labels are descriptive shorthand for the observed centroid patterns and are not validated phenotypes.

**Table 8:** Covariate-adjusted mediation analysis (Hayes PROCESS Model 4; 5000 bootstrap resamples). Adjusted for age, sex, sector, professional role, and prior AI training. Estimates are model-based associations derived from cross-sectionally measured variables; the terms “direct”, “indirect”, and “mediation” denote a statistical decomposition of covariance and do not denote demonstrated causal effects or temporal ordering. Path coefficients are unstandardized regression weights (denoted β) expressed in the natural units of the variables entered into each equation: AIELI was entered as a standardized z-score (per +1 SD), whereas ROI confidence and willingness to invest were entered on their original 1–5 Likert scales. Consequently, the a-path (AIELI → ROI confidence) is the change in ROI-confidence points per +1 SD of AIELI, the b-path is the change in willingness-to-invest points per one ROI-confidence point, and the indirect effect is in willingness-to-invest points per +1 SD of AIELI. Because the predictors are on different scales, these coefficients are not mutually comparable in magnitude and are not fully standardized β weights.

Path	Estimate (β)	SE	*p*-Value	95% Bootstrap CI
a: AIELI → ROI confidence	0.43	0.08	<0.001	—
b: ROI confidence → willingness to invest	0.31	0.09	0.001	—
c′: AIELI → willingness (direct)	0.17	0.07	0.018	—
c: AIELI → willingness (total)	0.30	0.08	<0.001	—
Indirect effect (a × b), bootstrapped (B = 5000)	0.13	—	—	[0.058, 0.231]
Proportion mediated	44.3%	—	—	—

**Table 9:** Moderated mediation analysis (Hayes PROCESS Model 7; sector as first-stage moderator; 5000 bootstrap resamples). Adjusted for age, sex, professional role, and prior AI training; sector entered as a first-stage moderator with public hospital as reference. Subgroup sizes: public *n* = 54, private *n* = 31, academic *n* = 23. Estimates are model-based associations from cross-sectional data and do not represent proven causal pathways. As in Table 8, path coefficients are unstandardized regression weights (β) in the natural units of the entered variables: AIELI is standardized (per +1 SD), while ROI confidence and willingness to invest remain on their 1–5 scales, so the conditional indirect estimates are expressed in willingness-to-invest points per +1 SD of AIELI within each sector. The index of moderated mediation is the difference between two such conditional indirect estimates and is reported in the same units.

Path/Effect	Estimate (β)	SE	*p*-Value	95% Bootstrap CI
a_1_: AIELI → ROI confidence (public hospital)	0.23	0.10	0.024	—
a_2_: AIELI → ROI confidence (academic)	0.45	0.13	<0.001	—
a_3_: AIELI → ROI confidence (private clinic)	0.62	0.11	<0.001	—
b: ROI confidence → willingness to invest	0.31	0.09	0.001	—
Conditional indirect: public	0.072	—	—	[0.014, 0.158]
Conditional indirect: academic	0.141	—	—	[0.043, 0.267]
Conditional indirect: private clinic	0.193	—	—	[0.087, 0.318]
Index of moderated mediation (private vs. public)	0.061	—	—	[0.012, 0.131]
AIELI × academic (vs. public), first-stage interaction	0.22	0.13	0.093	[−0.03, 0.47]
AIELI × private (vs. public), first-stage interaction	0.39	0.12	0.001	[0.15, 0.63]
Index of moderated mediation (academic vs. public)	0.034	—	—	[−0.006, 0.086]
Index of moderated mediation (private vs. academic)	0.027	—	—	[−0.011, 0.079]
AIELI × sector interaction (overall)	—	—	0.014	—

## Data Availability

The data presented in this study are available on request from the corresponding author. The dataset is not publicly available due to privacy and institutional ethics restrictions.
